# Ca^2+^-dependent Structural Changes in the B-cell Receptor CD23 Increase Its Affinity for Human Immunoglobulin E*[Fn FN2]

**DOI:** 10.1074/jbc.M113.480657

**Published:** 2013-06-17

**Authors:** Daopeng Yuan, Anthony H. Keeble, Richard G. Hibbert, Stella Fabiane, Hannah J. Gould, James M. McDonnell, Andrew J. Beavil, Brian J. Sutton, Balvinder Dhaliwal

**Affiliations:** From ‡ King's College London and the Medical Research Council and Asthma UK Centre in Allergic Mechanisms of Asthma, Randall Division of Cell and Molecular Biophysics, Guy's Campus, London, SE1 1UL and; the § Department of Biochemistry, University of Oxford, South Parks Road, Oxford OX1 3QU, United Kingdom

**Keywords:** Allergy, Calcium, FC Receptors, Immunology, X-ray Crystallography, CD23, Immunoglobulin E

## Abstract

Immunoglobulin E (IgE) antibodies play a fundamental role in allergic disease and are a target for therapeutic intervention. IgE functions principally through two receptors, FcϵRI and CD23 (FcϵRII). Minute amounts of allergen trigger mast cell or basophil degranulation by cross-linking IgE-bound FcϵRI, leading to an inflammatory response. The interaction between IgE and CD23 on B-cells regulates IgE synthesis. CD23 is unique among Ig receptors in that it belongs to the C-type (calcium-dependent) lectin-like superfamily. Although the interaction of CD23 with IgE is carbohydrate-independent, calcium has been reported to increase the affinity for IgE, but the structural basis for this activity has previously been unknown. We have determined the crystal structures of the human lectin-like head domain of CD23 in its Ca^2+^-free and Ca^2+^-bound forms, as well as the crystal structure of the Ca^2+^-bound head domain of CD23 in complex with a subfragment of IgE-Fc consisting of the dimer of Cϵ3 and Cϵ4 domains (Fcϵ3-4). Together with site-directed mutagenesis, the crystal structures of four Ca^2+^ ligand mutants, isothermal titration calorimetry, surface plasmon resonance, and stopped-flow analysis, we demonstrate that Ca^2+^ binds at the principal and evolutionarily conserved binding site in CD23. Ca^2+^ binding drives Pro-250, at the base of an IgE-binding loop (loop 4), from the *trans* to the *cis* configuration with a concomitant conformational change and ordering of residues in the loop. These Ca^2+^-induced structural changes in CD23 lead to additional interactions with IgE, a more entropically favorable interaction, and a 30-fold increase in affinity of a single head domain of CD23 for IgE. Taken together, these results suggest that binding of Ca^2+^ brings an extra degree of modulation to CD23 function.

## Teaser

**Background:** Immunoglobulin E (IgE) plays a central role in allergic disease. The B-cell receptor CD23 is pivotal in regulating IgE synthesis.

**Results:** Ca^2+^-dependent structural changes in CD23 enable additional interactions with IgE.

**Conclusion:** Mechanism for Ca^2+^-induced increase in affinity is revealed.

**Significance:** Ca^2+^ binding brings an extra degree of modulation to CD23 function.

## Introduction

The prevalence of allergy is increasing worldwide (1). Allergic diseases include asthma, hay fever, and anaphylactic shock and are commonly triggered by environmental allergens, resulting in an inflammatory response. Immunoglobulin E (IgE) antibodies play a central role in the mechanism of allergic disease (2), binding allergen via its Fab regions and interacting with two very different cell surface receptors, FcϵRI and CD23 (also known as FcϵRII), via its Fc region.

The FcϵRI receptor is primarily expressed on mast cells, basophils, and antigen-presenting cells and binds IgE with a high affinity (*K_D_* ∼10^−10^ m). It is responsible for allergic sensitization and the immediate type I hypersensitivity reaction in which minute amounts of allergen cross-link receptor-bound IgE on mast cells and basophils, triggering cellular degranulation (2).

The second principal receptor for IgE, CD23, is a type II transmembrane glycoprotein with a C-type lectin-like domain (CTLD)^4^ (3, 4). It is expressed in several hematopoietic cell types, including B-cells and antigen-presenting cells.

In its membrane-bound form, CD23 consists of three CTLD “heads” connected to the membrane via a trimeric α-helical coiled-coil “stalk” (5). The stalk region is susceptible to attack by various proteases, such as ADAM10 (6, 7), releasing soluble forms of CD23. The house dust mite allergenic protease *Der p* I generates a soluble monomeric form of CD23 consisting of just the lectin head domain, termed derCD23 (8, 9).

The interaction between CD23 and IgE is critical in the regulation of IgE synthesis, which can be up- or down-regulated depending on the oligomerization state of CD23 (2, 9, 10). The interaction between the proteins is also crucial in facilitated antigen presentation (11).

CD23 is the only Ig receptor that belongs to the CTLD superfamily (3, 4). Some CTLDs interact with carbohydrate moieties of glycoproteins via a bound Ca^2+^ ion, but other CTLD-containing proteins bind neither Ca^2+^ nor carbohydrate and interact with other ligands such as proteins (4, 12). Ca^2+^ and carbohydrate binding activities are limited to those CTLDs that contain the appropriate sequence motifs (3). These CTLDs contain between one and three Ca^2+^-binding sites as follows: site 2 is usually involved in carbohydrate binding, and site 1 and site 3 commonly play structural roles (4). Sequence analysis suggests that the CTLD of human CD23 has two putative Ca^2+^-binding sites: a conserved “principal” binding site (site 2 in CTLD nomenclature) and a less well conserved “auxiliary” binding site (site 1) (3, 13).

Human CD23 binds via its CTLD to each chain of the IgE dimer, in a carbohydrate-independent manner (14, 15). Although Ca^2+^ is not required (14), despite an early report that murine CD23 binding to IgE was Ca^2+^-dependent (16), enhanced binding of human CD23 in the presence of Ca^2+^ has been reported (17). However, the role played by Ca^2+^ in IgE binding has hitherto been unknown.

The structure of derCD23 has been determined previously, both by NMR (17) and x-ray crystallography (13). The solution structure suggests that Ca^2+^ binds at the auxiliary binding site, but the derCD23 crystal structure, incorporating a double mutation (H213R and G256S), indicates that a single Ca^2+^ ion binds at the principal binding site. Surprisingly, the crystal structure revealed that “loop 4,” known to contribute to IgE binding (18), is substantially disordered in the presence of Ca^2+^ and ordered in its absence. We recently solved the structure of derCD23 in complex with Fcϵ3-4 (a subfragment of IgE-Fc consisting of the dimer of Cϵ3 and Cϵ4 domains) and found loop 4 partially *disordered* in the absence of Ca^2+^ (15). There are other precedents for the stabilizing effects of Ca^2+^ upon loop 4 in CTLDs (4), such as mannose-binding lectin (19).

We have studied the binding of Ca^2+^ to wild-type, human derCD23, and its effect upon IgE binding using site-directed mutagenesis, x-ray crystallography, ITC, surface plasmon resonance, and stopped-flow fluorescence analysis. We demonstrate that a single Ca^2+^ ion binds at the principal binding site in the CTLD head domain of CD23, inducing a *trans* to *cis* isomerization of Pro-250 in loop 4 and concomitant conformational changes in the rest of the loop. The additional salt bridges and hydrogen bonds formed between IgE and CD23 upon Ca^2+^ binding give rise to a more entropically favorable interaction and a 30-fold increase in affinity.

## EXPERIMENTAL PROCEDURES

### 

#### 

##### Site-directed Mutagenesis of derCD23

Mutations (E249A, S252A, D258A, N269A, N269D, or D270A) were introduced into the derCD23 gene using the QuikChange Lightning site-directed mutagenesis kit (Stratagene). The wild-type derCD23 gene, in a pET5a vector (17), was used as a template for PCR. The primers for PCR incorporating the derCD23 mutations were synthesized by Sigma (see supplemental Table S1). The resulting vectors were transformed into BL21 (DE3) competent cells.

##### Expression, Refolding, and Purification of Proteins

Wild-type and six mutant derCD23 proteins were all expressed, refolded, and purified according to protocols previously described (15, 17). Correct folding of the derCD23 proteins was assessed by one-dimensional ^1^H NMR at 500 MHz (large dispersion and strong signals of methyl groups between 1.0 and −1.0 ppm).

IgE-Fc (including domains Cϵ2, Cϵ3, and Cϵ4) and Fcϵ3-4 were prepared as described previously (15).

##### Surface Plasmon Resonance

Experiments were carried out on a Biacore T200 instrument (GE Healthcare). Specific binding surfaces were prepared by coupling derCD23 to a CM5 sensor chip via amine coupling. Coupling density was limited to <100 RU. Samples of Fcϵ2-4 (IgE-Fc) were dialyzed into either HBS/Ca (10 mm HEPES, pH 7.4, 150 mm NaCl, 4 mm CaCl_2_, 0.01% (v/v) surfactant P-20) or HBS/EDTA (10 mm HEPES, pH 7.4, 150 mm NaCl, 10 mm EDTA, 0.01% (v/v) surfactant P-20) and injected over the sensor chip at a flow rate of 25 μl min^−1^ at concentrations of 4000, 2000, 1000, 500, 250, 125, 62.5, 31.2, 15.6, 7.8, and 3.9 nm. Each data set was collected at 5, 15, 25, and 35 °C. Analyte concentrations were injected in duplicate, a titration series of “low-to-high” analyte concentration followed by a “high-to-low” series. A 2-min association phase was followed by a 5-min dissociation phase, which allowed complete dissociation of analyte from the senor surface. Standard double-referencing data subtraction methods were applied (20) before evaluation of equilibrium binding. Data were analyzed using curve-fitting software from Microcal Origin (OriginLab).

##### Crystallization

All crystals were obtained by the hanging drop vapor diffusion method at 291 K and grew to their maximal size within 2 weeks.

All of the derCD23 crystals were grown by mixing protein at 3.5 mg/ml in 25 mm Tris, pH 7.5, and 137 mm NaCl with an equal volume of reservoir solution. Wild-type and S252A mutant derCD23 crystals were obtained using 18.5% PEG 6000, 2% 1,6-hexanediol, 0.05 m ammonium sulfate, and 0.1 m sodium acetate, pH 4.7, as the reservoir solution. E249A mutant derCD23 crystals were grown using 0.2 m potassium thiocyanate, 0.1 m Bistris propane, pH 8.5, and 20% PEG 3350. Crystals of D258A derCD23 were obtained using 18% ethanol, 4% PEG 400, and 0.1 m sodium acetate, pH 5.0. D270A mutant derCD23 crystals were formed using 30% PEG 4000 and 0.3 m ammonium sulfate as the reservoir. The derCD23 crystals were cryoprotected by soaking in reservoir solution containing an additional 20% glycerol, and they were subsequently flash-cooled in liquid nitrogen.

Ca^2+^ was bound to wild-type derCD23 by soaking the crystals with 25% PEG 6000, 2% 1,6-hexanediol, 0.1 m MES, pH 7.1, 10 mm CaCl_2_, and 15% glycerol for 3 days. The crystals were then flash-cooled.

Crystals of the Ca^2+^-bound derCD23-Fcϵ3-4 complex were grown using 0.22 mm Fcϵ3-4 (11 mg/ml) and 0.44 mm derCD23 (6.8 mg/ml, in 20 mm Tris 7.5, 20 mm NaCl and 0.05% sodium azide) diluted with an equal volume of 0.2 m sodium acetate trihydrate, 0.1 m Tris, pH 8.5, 16% PEG 4000, and 10 mm CaCl_2_, and the drops were then micro-seeded with crystals of the “apo” Fcϵ3-4-derCD23 complex (15). The crystals were flash-cooled using 0.2 m sodium acetate trihydrate, 0.1 m Tris, pH 7.5, 16% PEG 4000, 15 mm CaCl, and 25% glycerol as cryoprotectant.

##### Crystallographic Data Collection and Processing

All diffraction data were collected at 100 K at the Diamond Light Source (Harwell, UK) or Daresbury SRS (Cheshire, UK) synchrotrons. Indexing, integration, and merging of data were carried out with the *MOSFLM* (21, 22) or *HKL2000* (23) suite of programs.

##### Crystallographic Structure Determination

The derCD23 and Ca^2+^-bound derCD23- Fcϵ3-4 complex structures were solved by molecular replacement with *PHASER* (24) using the Ca^2+^-free derCD23 (PDB 2H2R) (13) and the derCD23- Fcϵ3-4 complex structures (PDB 4EZM) (15), respectively, as search models.

In the presence of a high degree of noncrystallographic symmetry, reflections were selected for the *R*_free_ set in thin resolution shells (25) using *SFTOOLS* (26). Iterative cycles of refinement using *REFMAC5* (27), *PHENIX* (28), and *BUSTER-TNT* (29) alternated with manual model building with *COOT* (30). During initial refinement, tight noncrystallographic symmetry restraints were used. These were gradually relaxed, and finally local structure similarity restraints (“local noncrystallographic symmetry”) (27, 29) were applied. TLS groups (31) were generated using the TLSMD web server (32). The crystallographic processing and refinement statistics are presented in Table 1.

In the Ca^2+^-bound derCD23-Fcϵ3-4 complex crystal structure, no electron density was observed for the following: Fcϵ3-4 residues 387–388 of chain A; 362–364 of chain B; 367–371 and 421–428 of chain C; 365–367, 387–389, 454–458, 462–463, 481–483, and 525–526 of chain D; 367–370, 419–422, 429–430, 446–449, 479–480, and 510–519 of chain E; and 363–364 and 446–450 of chain F. Therefore, these residues were not built into the model. Carbohydrate atoms and six Ca^2+^ ions were subsequently incorporated into the structure. Because of the low resolution of the Ca^2+^-bound derCD23-Fcϵ3-4 complex data, *B*-factor sharpening (27, 28, 30) was used to improve the quality of the electron density maps, thereby helping to resolve the positions of the Ca^2+^ ions. All of the structural figures presented were generated using PyMOL (33).

##### Isothermal Titration Calorimetry

Wild-type and mutant derCD23 protein samples were initially dialyzed against 10 mm Tris, pH 6.8, 125 mm NaCl, 10 mm EDTA to remove the bound Ca^2+^ present from the protein refolding procedure. This was followed by extensive dialysis against 10 mm Tris, pH 6.8, 125 mm NaCl buffer pretreated with 50 g/liter Chelex 100. Freshly prepared 20 mm CaCl_2_ in the same batch of assay buffer used to dialyze the protein was titrated into 50 μm derCD23 proteins at 25 °C in an iTC200 microcalorimeter (Microcal, GE Healthcare). Data were analyzed using the Microcal Origin software supplied with the machine using a 1:1 binding model under “low *c*-value” conditions where the stoichiometry is fixed at 1:1 (as shown by the crystal structure), and the *K_D_* and Δ*H* values were floated.

##### Stopped-flow Analysis

Experiments were carried out using a single-mix SF-61SX stopped-flow fluorimeter (TgK Scientific Ltd.) with a 1:1 mixing ratio. Experiments were carried out at 25 °C in the same buffer as for the ITC (see above) with 2 μm apo-derCD23 mixed against 20 mm CaCl_2_ with or without preincubation with stoichiometric amounts of the prolyl isomerase cyclophilin A. Tryptophan fluorescence was excited at 296 nm and all fluorescence emission collected above 305 nm. Typically, 6–10 runs were averaged before fitting to a double exponential (Equation 1) using the manufacturer's analysis software,

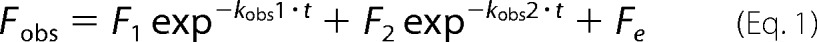
 where *F*_obs_ is the observed fluorescence change; *F_n_* is the fluorescence amplitude for the *n*th transient; *k*_obsn_ is the observed rate constant for the *n*th transient, and *F_e_* is the end point fluorescence.

## RESULTS

### 

#### 

##### Binding Affinities and Thermodynamics for the IgE-Fc/derCD23 Interaction Are Strongly Affected by Calcium

**FIGURE 1.**
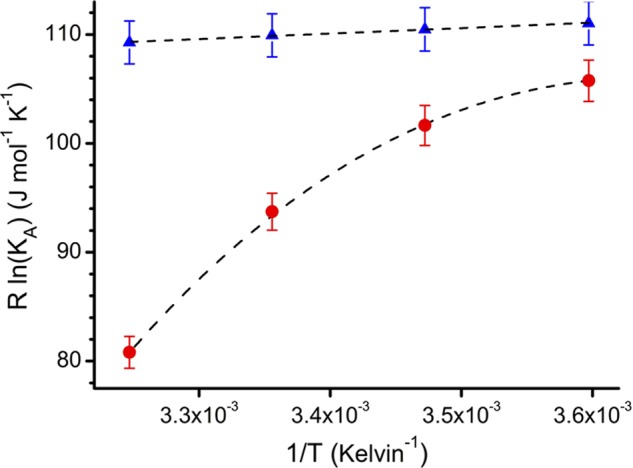
**Temperature dependence of the derCD23/IgE-Fc interaction in the presence and absence of Ca^2+^.** For Ca^2+^-bound and Ca^2+^-free states, binding affinities were measured by surface plasmon resonance at 5, 15, 25, and 35 °C; affinities at these temperatures for the Ca^2+^-bound states were 1.6, 1.7, 1.8, and 2.0 μm, respectively, and for the Ca^2+^-free state 3.0, 4.4, 10.2, and 58.0 μm, respectively. Data are shown as a van't Hoff plot (47) and were fitted to linear or nonlinear integrated forms of the van't Hoff equation (48). Ca^2+^-bound derCD23 (*blue triangles*) binds with a higher affinity than Ca^2+^-free derCD23 (*red circles*) to IgE-Fc. Ca^2+^-bound derCD23 shows small decreases in affinities at increasing temperatures, indicating a small favorable enthalpic contribution to binding energy (Δ*H* = −5.1 kJ mol^−1^). Ca^2+^-free derCD23 shows a larger temperature dependence, an indication of a larger contribution from enthalpy to the binding event (Δ*H* = −33.2 kJ mol^−1^); this interaction also shows a nonlinear temperature dependence, characteristic of an associated heat capacity change (Δ*Cp*).

The binding affinity of IgE-Fc for derCD23 is increased in the presence of Ca^2+^. The difference in binding affinity is highly temperature-dependent. At 5 °C, the difference in affinity is less than 2-fold (1.6 and 3.0 μm), but at 35 °C there is a 30-fold difference in binding affinity (2 and 58 μm) (Fig. 1 and supplemental Fig. S1). The thermodynamic parameters of the two interactions are also notably different, as shown in a van't Hoff plot of the binding thermodynamics (Fig. 1). In the presence of Ca^2+^, the IgE-Fc/derCD23 affinity shows only a small temperature dependence; for this interaction, a small favorable enthalpic contribution to binding is observed (Fig. 1), but the binding is largely driven by a favorable entropic term. In contrast, in the absence of calcium, the binding affinity of IgE-Fc/derCD23 is highly temperature-dependent (Fig. 1), consistent with a binding event largely driven by favorable enthalpy. The calcium-free binding event also shows a notable nonlinearity in the van't Hoff plot, indicating a significant Δ*Cp* component for this interaction (Fig. 1).

##### Comparison of Ca^2+^-free and Ca^2+^-bound derCD23 Crystal Structures

The Ca^2+^-free and Ca^2+^-soaked derCD23 crystals both belong to space group *P*1 and have four molecules within the asymmetric unit (Table 1). Although the unit cells index differently, the derCD23 molecules pack in an almost identical arrangement in both crystal lattices.

**TABLE 1:** **Crystallographic data processing and refinement statistics**

	Ca^2+^-bound derCD23	Ca^2+^-free derCD23	E249A derCD23	S252A derCD23	D258A derCD23	D270A derCD23	Ca^2+^-bound derCD23-Fcϵ3-4
PDB accession code	4G9A	4G96	4GI0	4GJ0	4GJX	4GK1	4GKO

**Data processing statistics**							
Beamline	DLS I03	Daresbury 14.1	DLS I03	DLS I02	DLS I03	DLS I02	DLS I04-1
Wavelength	0.9763 Å	1.4880 Å	0.9763 Å	0.9795 Å	0.9763 Å	0.9795 Å	0.9173 Å
Processing software	HKL2000	HKL2000	HKL2000	HKL2000	MOSFLM	HKL2000	MOSFLM
Space group	*P*1	*P*1	*P*4_1_22	*P*1	*P*6_5_	*P*2_1_2_1_2_1_	*P*2_1_2_1_2_1_
Unit cell parameters	*a* = 54.24 Å	*a* = 52.40 Å	*a* = 63.32 Å	*a* = 52.50 Å	*a* = 90.97 Å	*a* = 73.78 Å	*a* = 62.83 Å
	*b* = 53.71°	*b* = 56.73°	*b* = 63.32°	*b* = 56.87°	*b* = 90.97°	*b* = 109.11°	*b* = 110.13°
	*c* = 56.64°	*c* = 62.20°	*c* = 261.42°	*c* = 62.68°	*c* = 351.68°	*c* = 139.00°	*c* = 367.41°
	α = 112.92°	α = 68.49°		α = 68.06°			
	β = 88.53°	β = 88.16°		β = 88.23°			
	γ = 114.96°	γ = 73.40°		γ = 73.53°			
No. of mol/a.u.	4	4	3	4	8	7	9
Resolution range*^a^*	50.0 to 2.00 Å (2.07 to 2.00 Å)	57.2 to 2.25 Å (2.37 to 2.25 Å)	65.4 to 2.27 Å (2.40 to 2.27 Å)	50.0 to 1.95 Å (2.02 to 1.95 Å)	58.67 to 2.80 Å (2.95 to 2.80 Å)	50.0 to 2.25 Å (2.33 to 2.25 Å)	73.5 to 3.30 Å (3.48 to 3.30 Å)
Observations	139,591	82,009	323,278	97,210	133,827	382,280	157,453
Unique reflections	34,591	28,279	25,713	45,157	36,967	54,601	33,757
Average redundancy	4.0 (4.1)	2.9 (2.9)	12.6 (13.0)	2.2 (2.1)	3.6 (3.5)	7.0 (6.7)	4.7 (4.9)
Completeness	97.9% (96.9%)	94.6% (86.7%)	100% (100%)	97.1% (93.7%)	91.6% (94.3%)	99.7% (99.5%)	84.0% (86.3%)
Wilson *B*-factor	20.0 Å^2^	42.5 Å^2^	50.7 Å^2^	29.9 Å^2^	41.5 Å^2^	37.8 Å^2^	92.9 Å^2^
*I*/σ(*I*)	20.5 (4.6)	10.8 (3.6)	12.9 (2.3)	20.0 (2.0)	7.0 (1.9)	34.0 (4.0)	5.1 (1.4)
*R*_merge_*^b^*/*R*_p.i.m._*^c^*	0.096*^b^* (0.406)	0.065*^b^* (0.226)	0.020*^b^* (0.359)	0.049*^b^* (0.369)	0.081*^c^* (0.400)	0.078*^b^* (0.463)	0.089*^c^* (0.501)

**Refinement statistics**							
Resolution range	38.8 to 2.00 Å	39.4 to 2.25 Å	65.6 to 2.27 Å	31.3 to 1.95 Å	78.8 to 2.80 Å	43.9 to 2.24 Å	183.7 to 3.30 Å
Total no. of reflections	34,538	26,856	24,325	45,141	35,034	54,533	31,969
No. of working reflections	32,806	25,433	23,019	42,853	33,200	51,765	30,285
No. of test reflections	1732	1423	1306	2288	1834	2768	1684
*R* factor*^d^*	0.155	0.176	0.207	0.169	0.210	0.183	0.275
*R*_working_	0.152	0.173	0.205	0.166	0.208	0.181	0.272
*R*_free_	0.205	0.236	0.251	0.212	0.251	0.226	0.312
No. of atoms	4,697	4,571	3,328	4,710	8,810	8,120	16,217
Protein	4,271	4,273	3,202	4,257	8,635	7,452	15,823
Others*^e^*	426	298	126	453	175	668	394
r.m.s. bond length deviation	0.006 Å	0.017 Å	0.010 Å	0.007 Å	0.006 Å	0.006 Å	0.020 Å
r.m.s. bond angle deviation	0.963°	1.944°	1.375°	1.031°	0.987°	0.937°	1.963°
Mean *B* factor	25.7 Å^2^	41.3 Å^2^	31.6 Å^2^	40.4 Å^2^	51.0 Å^2^	43.5 Å^2^	150.5 Å^2^
Main chain	22.3	39.1	30.5	37.0	50.5	40.8	148.8
Side chain	29.1	43.6	32.6	43.7	51.5	46.1	152.1
Others*^e^*	35.5	44.4	46.3	48.2	32.5	53.5	163.6
r.m.s. backbone *B* factor deviation*^f^*	2.4	2.0	1.1	2.9	1.0	2.3	3.5
Ramachandran statistics*^g^*							
Favored	96.2%	94.1%	93.7%	96.0%	95.3%	95.5%	90.9%
Allowed	100%	99%	99.5%	99.8%	99.9%	100%	99.1%
Outliers	0%	1%	0.5%	0.2%	0.1%	0%	0.9%

*^a^* Values in parentheses are for the outer resolution shell.

*^b^ R*_merge_ = Σ|*I*_obs_ − 〈*I*〉|/Σ〈*I*〉.

*^c^ R*_p.i.m._ (precision-indicating merging *R*-factor) = Σ*_hkl_*(1/(*N* − 1))^1/2^ Σ*_i_*|*I_i_*(*hkl*) − *I*(−*h*−*k*−*l*)|/Σ*_hkl_*Σ*_i_I_i_*(*hkl*) (49).

*^d^ R* factor = Σ*_hkl_*‖*F_o_*(*hkl*)| − |*F_c_*(*hkl*)‖/Σ*_hkl_*|*F_o_*(*hkl*)|.

*^e^* Ca^2+^ ions, carbohydrate, water molecules, SO_4_^2−^ ions and glycerol.

*^f^* Root mean square (r.m.s.) deviation between *B*-factors for bonded main chain atoms.

*^g^* Data are defined by MolProbity (50).

The CTLD topology of all the derCD23 molecules is essentially identical, with Cα root mean square deviations ranging from 0.2 to 1.2 Å (over 120 Cα pairs) (for individual pairwise comparisons see supplemental Table S2). The structure consists of two α-helices and two β-sheets formed by eight β-strands with four disulfide bonds (Cys residues 160–288, 163–174, 191–282, and 259–273). Unlike previously solved derCD23 crystal structures (PDB codes 2H2R and 2H2T (13)), electron density for all the head domain is well observed, including loop 1 (Leu-226 to Glu-231) and loop 4 (Arg-253 to Glu-257) following earlier terminology (13). These loops are known to contribute to IgE binding (15, 18).

**FIGURE 2.**
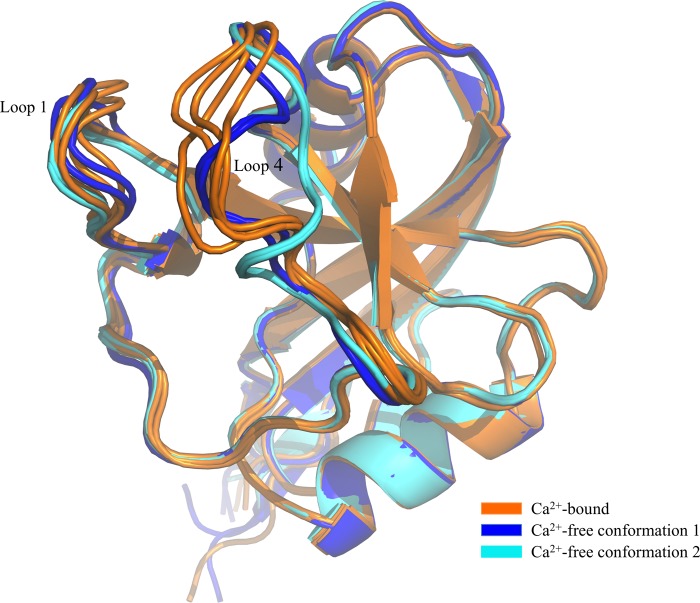
**Comparison of Ca^2+^-bound and Ca^2+^-free derCD23 structures.** Superimposition of Ca^2+^-free derCD23 structures (colored *blue* and *cyan*) and Ca^2+^-bound derCD23 structures (*orange*) is shown. Many of the residues that undergo conformational change upon calcium binding are also involved in the interaction with IgE.

**FIGURE 3.**
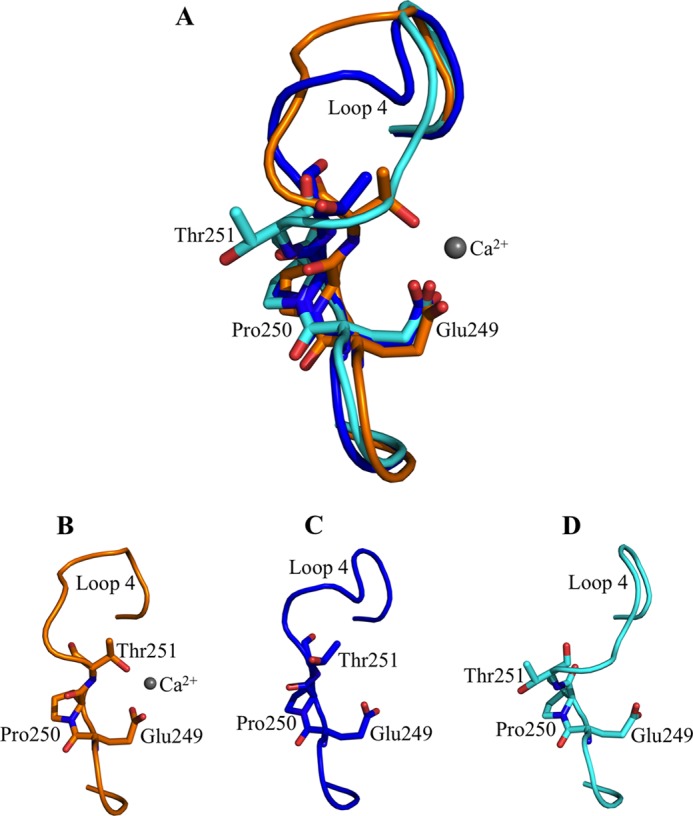
**Comparison of the Glu-249–Pro-250–Thr-251 (EPT) motif in derCD23 structures.** *A,* superimposition of the EPT motif in Ca^2+^-bound derCD23 (*orange*) and two conformations of Ca^2+^-free derCD23 structures (*blue* and *cyan*). In the Ca^2+^-bound and Ca^2+^-free conformation 1 derCD23 structures, Pro-250 adopts a *cis*-configuration. Pro-250 adopts a *trans*-configuration in conformation 2 of the Ca^2+^-free derCD23 structure. *B,* EPT motif in the Ca^2+^-bound derCD23 structure. *C,* EPT motif in conformation 1 of the Ca^2+^-free derCD23 structure. *D,* EPT motif in conformation 2 of the Ca^2+^-free derCD23 structure.

Comparison of the Ca^2+^-free and Ca^2+^-bound derCD23 structures reveals that the only conformational differences are in loop 4 and, to a lesser degree, in loop 1 (Fig. 2). Although the four copies of loop 4 in the Ca^2+^-bound structure adopt similar conformations, two copies of this loop in the Ca^2+^-free structure adopt a different conformation, and the other two adopt yet another (Fig. 2). These two different conformations of Ca^2+^-free derCD23 are not the result of different crystal packing interactions. In Ca^2+^-free “conformation 1,” two principal binding site residues (Glu-249 and Asp-270) are in a similar position to that observed in the Ca^2+^-bound structure, although the orientation of the Thr-251 side chain is different. In Ca^2+^-free “conformation 2,” only Glu-249 remains in a similar position when compared with the Ca^2+^-bound structure, although marked differences are seen for both Thr-251 (Fig. 3) and Asp-270.

Residues 249–251 in particular (the Glu-Pro-Thr, EPT, motif) are distinctly different between the Ca^2+^-bound and Ca^2+^-free derCD23. In the Ca^2+^-bound structure, Pro-250 adopts a *cis* configuration in all four copies (Fig. 3), and the distance between Thr-251 (Oγ1) and Glu-249 (Oϵ1) is 3.7 Å. In the Ca^2+^-free structure, this distance is 6.1 Å in conformation 1 and 8.3 Å in conformation 2; Pro-250 is *cis* in the former and *trans* in the latter.

These two possible configurations of Pro-250 in the absence of Ca^2+^ have an effect upon adjacent residues. The *trans* configuration of Pro-250 forms two hydrogen bonds with Arg-253, whereas *cis* Pro-250 cannot; consequently, both Arg-253 and Ser-254 are disordered when Pro-250 is *cis* (conformation 1), but are ordered, with Arg-253 pointing into a cavity between loops 1 and 4 (supplemental Fig. S2), when Pro-250 is *trans* (conformation 2). In the presence of Ca^2+^, Pro-250 is effectively locked into the *cis* configuration.

##### Ca^2+^ Binds in the Principal Evolutionarily Conserved Binding Site of CD23

**FIGURE 4.**
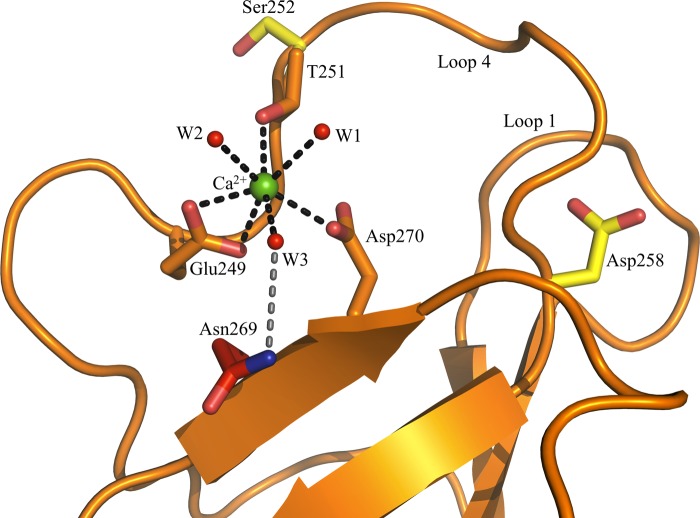
**Ca^2+^ binding to derCD23 as seen in three of the four molecules in the asymmetric unit (molecules A, B, and C).** Ca^2+^ binding to the principal binding site is as follows: three residues (Glu-249, Thr-251, and Asp-270) provide four oxygen atoms, and three water molecules provide three oxygen atoms (*black dashed lines*). Asn-269 is not directly involved in the binding of Ca^2+^, but it forms a H-bond with a ligating water molecule (*gray dashed line*). The auxiliary site residues Ser-252 and Asp-258 are distant from one another.

Unlike MBL (34) and DC-SIGN (35) that bind two and three Ca^2+^ ions, respectively, Ca^2+^-soaked human derCD23 crystals reveal that a single Ca^2+^ ion binds at the principal binding site in all four molecules of the asymmetric unit (Fig. 4). The electron density for the Ca^2+^-coordinating ligands (Glu-249, Thr-251, Asp-270, and three water molecules) and the Ca^2+^ ion itself is well defined in three of the derCD23 molecules; electron density for the three water molecules and Thr-251 in the fourth molecule is unclear. Although the site of Ca^2+^ binding is consistent with the observations of Wurzburg *et al.* (13), the details of the coordination of Ca^2+^ differ from that reported by them for the double mutant (H213R/G256S) human derCD23 crystal structure (PDB code 2H2T).

In the wild-type Ca^2+^-bound derCD23 crystal structure presented here, three residues (Glu-249, Thr-251, and Asp-270) provide four oxygen atoms, and three water molecules provide a further three for the binding of Ca^2+^ (Fig. 4). The coordination geometry is pentagonal bipyramidal formed by the seven ligands. This differs from the pentagonal bipyramidal coordination with one bisected apex formed by eight ligands, as seen in MBL (34), DC-SIGN (35), and human asialoglycoprotein receptor-1 (ASGPR-I) (36).

Surprisingly, in contrast to Wurzburg *et al.* (13), we found that the highly conserved Asn-269 residue (3, 13) is not directly involved in the binding of Ca^2+^; the nitrogen atom of the carboxamide group was found to form a hydrogen bond with a water molecule that acts as a Ca^2+^ ligand (Fig. 4). Additionally, we found that three water molecules, not two as previously suggested (13), directly ligate Ca^2+^.

To verify that the principal site is indeed the location of Ca^2+^ binding to derCD23 in solution, and to confirm the exact nature of the interaction, several single point mutations in derCD23 were generated: conserved residues (3, 13) were mutated within the principal (E249A and D270A) and auxiliary (S252A and D258A) calcium-binding sites, along with the highly conserved Asn-269 residue (N269A and N269D). The ability of wild-type and mutant derCD23 to bind calcium was assessed by ITC (Table 2 and supplemental Fig. S3).

**TABLE 2:** **Binding affinities of Ca^2+^ for wild-type or mutant derCD23 studied by ITC** Mutants E249A, D258A, and D270A showed no measurable Ca^2+^ binding.

Ca^2+^-binding site	Construct	Binding affinity (*K_D_*)
	Wild type derCD23	1.5 mm
Principal	E249A	Not measurable
Principal	D270A	Not measurable
Auxiliary	S252A	1.5 mm
Auxiliary	D258A	Not measurable
	N269A	0.73 mm
	N269D	1.5 mm

Wild-type derCD23 was found to have a *K_D_* for Ca^2+^ of 1.5 mm. This agrees with a previously calculated affinity determined by NMR perturbation studies (17). As expected, the two principal binding site derCD23 mutants, E249A and D270A, were unable to bind Ca^2+^.

As predicted from the crystal structure, the introduction of the S252A mutation did not affect Ca^2+^ binding, but unexpectedly, the D258A mutant was unable to bind Ca^2+^ even though the aspartate forms part of the apparently nonfunctioning auxiliary Ca^2+^-binding site.

The N269A and N269D mutants retained the ability to bind Ca^2+^, consistent with our observation that Asn-269 is not directly involved in Ca^2+^ ligation. In fact, the affinity of the N269A mutant for Ca^2+^ is 2-fold higher.

##### Principal and Auxiliary Ca^2+^-binding Site Mutant derCD23 Structures

The crystal structures of the derCD23 principal and auxiliary Ca^2+^-binding site mutants were determined (but not those of N269A or N269D mutants) (supplemental Fig. S4).

The S252A auxiliary binding site mutant crystallized in the same conditions and same crystal form as wild-type Ca^2+^-free derCD23. Similarly, Pro-250 is *trans* in two copies and *cis* in the others. D258A (auxiliary) and D270A (principal site) crystallized differently, and in all eight and seven molecules within the asymmetric unit, respectively, Pro-250 is *trans*. In contrast the E249A (principal site) mutant, which crystallized in yet another form, has *cis* Pro-250 in all three independent copies. Here also, loop 4 adopts a similar conformation to that observed in the Ca^2+^-bound derCD23 structure (and conformation 2 of the Ca^2+^-free and S252A structures). Mutation of the adjacent Glu-249, part of the EPT motif, to alanine appears to have locked Pro-250 into the *cis* configuration. Thus, of the 30 independent copies of derCD23 presented here, 19 are *trans*; of the remaining 11, four are *cis* as a result of the Ca^2+^ present, and 3 are *cis* as a result of mutation of the adjacent Glu-249. Thus, *trans* is apparently favored in the absence of Ca^2+^ or other factors.

No major conformational changes were observed in either the principal or auxiliary Ca^2+^-binding sites upon introduction of the point mutations that could inadvertently affect the binding of Ca^2+^, with the exception of D258A. Mutation of Asp-258, which faces Pro-250 at the base of loop 4, to alanine results in a conformational change in loop 4 and a reorientation of Arg-253 compared with the Ca^2+^-free derCD23 structure; this was seen in all eight molecules within the asymmetric unit. The side chain of Arg-253 flips ∼180° and projects into the principal Ca^2+^-binding site cavity, occupying the Ca^2+^ ion position (supplemental Fig. S5). The re-positioned Arg-253 side chain forms two salt bridges with the carboxylate group of Glu-249. Occupation of the Ca^2+^-binding site by Arg-253 explains why the D258A mutant fails to bind Ca^2+^.

Intriguingly, in the previously determined Ca^2+^-free crystal structure of the double-mutant derCD23 (13), loop 4 becomes ordered in the *absence* of Ca^2+^ because of a similar rearrangement of the side chain of Arg-253, which again occupies the Ca^2+^-binding site. In that study, however, the conformation of loop 4 and the orientation of Arg-253 is likely due to the nonsilent G256S mutation that was incorporated into loop 4 (13).

##### Ca^2+^-induced Conformational Changes Are Accelerated by Prolyl Isomerase Cyclophilin A

**FIGURE 5.**
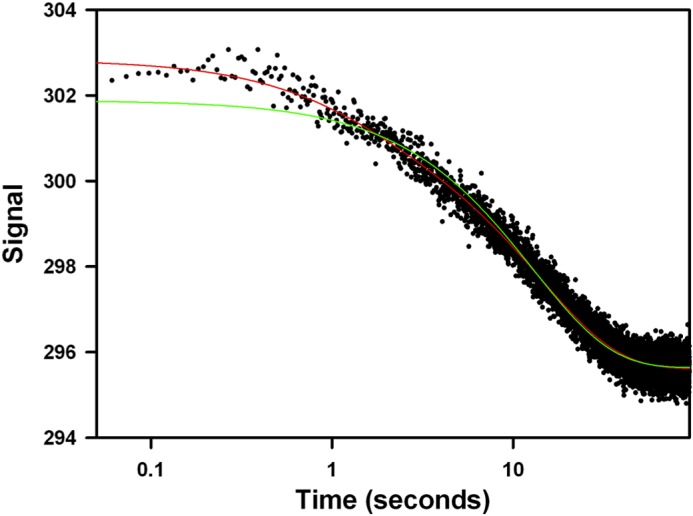
**Transient kinetics experiments reveal that Ca^2+^ binds to derCD23 through a two-step mechanism.** Mixing 20 mm CaCl_2_ with 2 mm derCD23 in assay buffer results in a biphasic fluorescence quench, with a rapid burst-like phase followed by a slower and larger amplitude transient. The *red* and *green lines* show fits to the data of double and single exponential equations, respectively; the latter fitting is significantly worse, especially over the shorter times. The *x* axis (time) is shown on a logarithmic scale as the two phases occur over very different time ranges. The *y* axis is the fluorescence amplitude in arbitrary units.

Stopped-flow kinetic analysis of changes in protein tryptophan fluorescence has previously been used to identify the presence of prolyl *trans-cis* isomerization accompanying Ca^2+^ binding to C-type lectins (37). To determine whether this was occurring with derCD23, Ca^2+^-free derCD23 and Ca^2+^ were mixed in a stopped-flow experiment (Fig. 5). This revealed biphasic kinetics as follows: an initial fast but low amplitude quench followed by a slower quench that dominated the overall fluorescence change. Comparison with studies of MBL (37) suggests that the fast phase reports on the Ca^2+^-binding step and the slower phase the prolyl isomerization. Consistent with this is the observation that repeating the experiment in the presence of cylophilin A, a prolyl isomerase, accelerated the observed rate constant for the slower step (supplemental Fig. S6), as seen for Ca^2+^ binding to MBL (37).

##### Ca^2+^-induced Ordered Loop 4 of derCD23 Makes Additional Interactions with IgE Fcϵ3-4

The Ca^2+^-bound derCD23-Fcϵ3-4 complex crystallized in essentially the same crystal form as the previously reported Ca^2+^-free complex (15), but with a 2% increase in the length of the *c*-axis. The packing arrangements of the molecules within the two lattices are virtually identical. Data processing and structural determination statistics for the Ca^2+^-bound complex are presented in Table 1.

**FIGURE 6.**
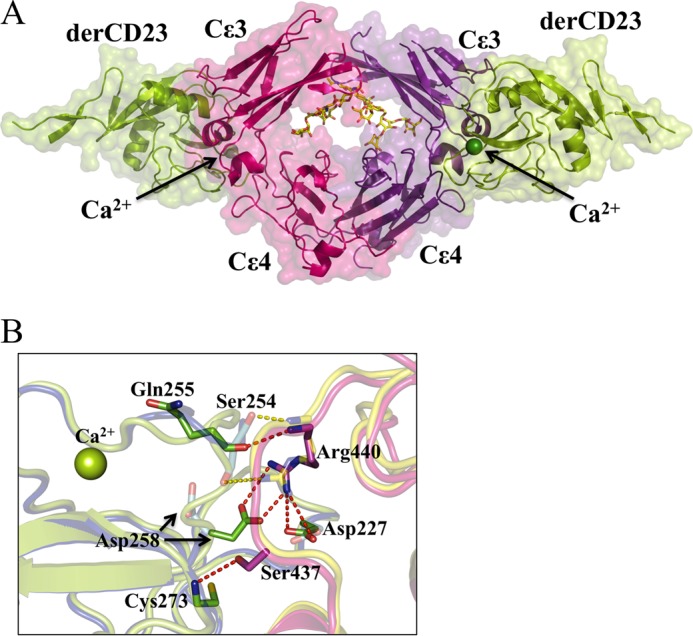
**Structure of the Ca^2+^-bound derCD23-Fcϵ3-4 complex.** *A,* two molecules of derCD23 (*green*, Cα traces with surfaces) bind one to each heavy chain of Fcϵ3-4 (*pink* and *purple*). The carbohydrate (*red, blue,* and *yellow*, in all-atom representation) and Ca^2+^ ions (*green spheres*) are also shown. *B,* enlarged view of a region of the Ca^2+^-bound and Ca^2+^-free derCD23-Fcϵ3-4 interfaces. In the presence or absence of Ca^2+^, the majority of interactions at the interface remain the same, only the *differences* are highlighted as follows: six hydrogen bonds (*red dashes*) are observed only in the Ca^2+^-bound form of the complex (Fcϵ3-4 in *pink*, derCD23 in *green*); two hydrogen bonds (*yellow dashes*) are seen only in the Ca^2+^-free form of the complex (Fcϵ3-4 in *blue*, derCD23 in *yellow*). Thus there is a net gain of four hydrogen bonds, mainly associated with the two new salt bridges between derCD23 and Fcϵ3-4 (Asp-227 to Arg-440 and Asp-258 to Arg-440) upon Ca^2+^ binding.

In the derCD23-Fcϵ3-4 complex structure, there are three independent complexes per asymmetric unit. The six heavy chains of Fcϵ3-4 (labeled A–F) are bound to six derCD23 molecules (labeled G–L). A single derCD23-Fcϵ3-4 complex (comprising dimeric Fcϵ3-4 chains C and D, bound to derCD23 head domains I and J) is depicted in Fig. 6.

Fcϵ3-4 is in a very “closed” conformation (15, 38) when bound to derCD23. The angle between the Cϵ3 and Cϵ4 domains in the Ca^2+^-bound derCD23-Fcϵ3-4 complex differ by only 5°, similar to the range observed in the Ca^2+^-free complex structure. This closed conformation allosterically precludes binding to FcϵRI (15, 39).

The derCD23 molecules within the Ca^2+^-bound derCD23-Fcϵ3-4 complex presented here are essentially identical, with Cα root mean square deviations ranging from 0.21 to 0.27 Å (over 120 Cα pairs). Each of the six derCD23 molecules has a Ca^2+^ ion bound at the principal binding site; the electron density for the ion is clear in four of the molecules but is ambiguous in copies H and K. Comparison of the derCD23 molecules from the Ca^2+^-free and Ca^2+^-bound complexes shows that the only substantial difference involves residues Ser-256 and Glu-257 of loop 4, for which no electron density was observed in the Ca^2+^-free complex (15). In contrast, electron density is observed for the entire loop, including residues Ser-256 and Glu-257, in all six copies of derCD23 in the Ca^2+^-bound complex.

Turning to the interface between derCD23 and Fcϵ3-4, which is almost identical in all six copies in the Ca^2+^-bound complex, there are a number of differences compared with the Ca^2+^-free complex (15), all involving loop 4 and its immediate vicinity (Fig. 6 and supplemental Fig. S7). All four salt bridges and two of the four additional hydrogen bonds observed in the Ca^2+^-free complex (15) are retained in the Ca^2+^-bound complex; the two hydrogen bonds lost are between Ser-254 (derCD23) and Arg-440 (Fcϵ3-4). However, in the Ca^2+^-bound complex, there are two additional salt bridges (both Asp-227 and Asp-258 with Arg-440) and two new hydrogen bonds formed (Gln-255 to Arg-440 and Cys-273 to Ser-437) (Fig. 6*B*). Arg-440, in the Cϵ3-Cϵ4 linker region, is key to these additional interactions with Asp-227 (loop 1) and Asp-258 (loop 4) following Ca^2+^-induced ordering and conformational change in loop 4.

This involvement of Asp-258 is particularly intriguing because it is the only conserved residue of the auxiliary binding site that remains in human CD23 (supplemental Fig. S8). Thus, although Ca^2+^ binds only at the principal binding site, it induces conformational changes that bring into play Asp-258 from the auxiliary site, which then makes additional contacts with IgE to enhance the affinity.

A final observation based upon inspection of conformational changes in loops 1 and 4 of both Ca^2+^-bound and -free complex structures suggests that the Ca^2+^ ion appears to be able to bind to CD23 either before or after engagement of IgE.

## DISCUSSION

Although CD23 recognizes IgE through its CTLD “head,” this interaction does not involve carbohydrate (14) and occurs in the absence of Ca^2+^, although Ca^2+^ substantially enhances the affinity (17). In mouse, the IgE/CD23 interaction is reported to be Ca^2+^-dependent (16), but murine CD23 may differ in its Ca^2+^-binding ability, since both the “principal” and “auxiliary” Ca^2+^-binding sites (with reference to MBL, which binds two Ca^2+^ ions (40)) are conserved (supplemental Fig. S7). Only the principal Ca^2+^ site is fully conserved in human CD23, but previous structural studies on soluble derCD23 had identified conflicting locations for the binding of a single Ca^2+^ ion. The NMR solution structure of derCD23, together with chemical shift perturbation measurements, suggested that Ca^2+^ binds only at the auxiliary binding site (17), but subsequent crystallographic analysis of a double mutant of derCD23 (H213R and G256S) showed Ca^2+^ bound solely at the principal site (13).

To resolve the controversy, the crystal structure of Ca^2+^-bound wild-type human derCD23 was solved. Several single point mutations in both the principal and auxiliary binding sites of derCD23 were also generated; their crystal structures were solved, and their ability in solution to bind calcium was assessed by ITC. The structure of Ca^2+^-bound derCD23 reported here shows a single ion bound at the principal calcium site, liganded by Glu-249, Thr-251, Asp-270, and three water molecules; the *K_D_* is 1.5 mm. Ca^2+^ binding to the mutant derCD23 molecules (E249A and D270A, principal; S252A and D258A, auxiliary) was consistent with binding only to the principal site, with the exception of D258A, which showed no Ca^2+^ binding (Table 2). However, the crystal structure of this mutant, in contrast to the other three structures that all showed perturbations only at the site of mutation, revealed that the D258A mutation (at the base of loop 4) caused a major reorientation of Arg-253 such that its guanidinium group occupied the principal Ca^2+^-binding site (supplemental Fig. S4). (A similar reorientation of Arg-253 was reported in the earlier double mutant derCD23 Ca^2+^-free crystal structure (13), caused there, presumably, by the G256S mutation at the center of loop 4).

Although the results presented here agree with the earlier crystal structure in terms of the location of the single Ca^2+^ ion (13), the nature of the coordination differs; the earlier study identified Asn-269 as a direct ligand, and the present study indicates that this conserved residue is not a ligand, although it forms a hydrogen bond to one of the Ca^2+^-ligating water molecules (Fig. 4). The noncritical role of Asn-269 was confirmed by the fact that the mutation N269D left Ca^2+^-binding unaffected, and the N269A mutation even enhanced affinity (Table 2); the direct involvement of Asn-269 in the earlier structure may be another consequence of the G256S mutation (13). The reason why the NMR analysis of derCD23 appeared to show Ca^2+^ binding at the auxiliary site (17) is probably due to the conformational changes that we observe in loop 1 upon Ca^2+^ binding to the principal site, but why changes in chemical shift were not observed in the principal Ca^2+^-binding site remains unclear.

The effect of Ca^2+^ binding upon the structure of derCD23 is limited to loops 1 and 4 only, although the latter shows greater changes (Fig. 2). In the presence of Ca^2+^_,_ all loop 4 conformations are essentially similar, and in the absence of Ca^2+^, the loop adopts either of two different structures. All residues of this loop are defined in both crystal structures, however, in contrast to the earlier double mutant derCD23 study in which the loop was ordered in the *absence* of Ca^2+^ but partially disordered in its presence (13). The isomerization state of Pro-250 appears to be a key determinant of the conformation of loop 4, which is found to be either *cis* or *trans* in the Ca^2+^-free structures, but it is locked in *cis* in Ca^2+^-bound CD23.

This is consistent with the extensive studies by Ng *et al.* (19, 37) showing that binding of Ca^2+^ at the principal site (site 2) of MBL is coupled to a conformational change in loop 4 that involves the *trans-cis* isomerization of Pro-191, the structural equivalent of Pro-250 in CD23. In the absence of Ca^2+^, MBL exists as a mixture of two conformers, but binding of Ca^2+^ traps loop 4 and Pro-191 in the *cis* form (19); this trapping only occurs when Ca^2+^ binds at the principal site (site 2 in MBL) and not when Ca^2+^ binds only to the auxiliary site (site 1 in MBL).

Weis and co-workers (37) complemented these structural studies by monitoring Ca^2+^ binding to MBL using stopped-flow spectrofluorimetry. This revealed a characteristic biphasic fluorescence change as follows: a fast (∼1 s^−1^) burst phase followed by a slower conformational change, identified as the *trans/cis* prolyl isomerization accompanying Ca^2+^ binding because the observed rate was accelerated in the presence of cyclophilin A.

We performed a similar stopped-flow spectrofluorimetry study of Ca^2+^ binding to derCD23, and we discovered striking similarities to the MBL results. First, there is a biphasic fluorescence change with observed rates for the two steps close to those seen for MBL (Fig. 5). Second, the observed rate for the slower phase is also accelerated in the presence of the prolyl isomerase cyclophilin A. This suggests that Ca^2+^ binding triggers a proline isomerization, presumably to the *cis* form seen in the Ca^2+^-bound structure.

The binding of Ca^2+^ to CD23 enables it to make additional interactions with IgE. In the structure of the complex between Ca^2+^-free derCD23 and Fcϵ3-4, the interface is dominated by four salt bridges and four additional hydrogen bonds (15). Loop 4, immediately adjacent to the interface, is disordered at residues Ser-256 and Glu-257. The structure of the complex that we now report here, in the presence of Ca^2+^, is virtually identical in all regards except for the structure of loop 4, which is fully ordered (Fig. 6). The consequence of this Ca^2+^-induced structural change is that two additional salt bridges are made, as well as two new hydrogen bonds, although two that were present in the Ca^2+^-free structure are lost (Fig. 6*B*). These additional salt bridges, Asp-227 and Asp-258 with Arg-440, which lie in the Cϵ3-Cϵ4 linker region, undoubtedly make the principal contribution to the enhanced affinity of CD23 for IgE in the presence of Ca^2+^.

The thermodynamic analysis of the interaction between derCD23 and IgE (Fig. 1) is consistent with the ordering effect of Ca^2+^ binding on loop 4. In the presence of calcium, the interaction is entropically favorable, and without calcium it is entropically unfavorable. Although there are undoubtedly significant differences in how water and counter ions behave in the Ca^2+^-bound and Ca^2+^-free structures, the most obvious structural effect is on the dynamics of loop 4. The greater entropic contribution to binding in the presence of Ca^2+^ therefore at least partly reflects the smaller entropic penalty upon forming the complex with a more ordered loop 4. The earlier NMR study of derCD23 showed that this loop exhibits substantial mobility when free in solution (17).

Although a single CD23 head domain binds to IgE with a *K_D_* of about 1.5 μm (this paper and see Ref. 41), compared with 10^−10^–10^−11^ m for FcϵRI, the avidity effect achieved when two or more CD23 heads of the trimeric molecule engage two or more IgE molecules in an immune complex or on the surface of a cell is considerable and may approach that of FcϵRI binding (9, 42–44). Thus, the 30-fold enhancement of CD23 binding to IgE in the presence of Ca^2+^ could have a profound effect when amplified through avidity.

Ca^2+^ binds to derCD23 with a *K_D_* of 1.5 mm. The physiological concentration of Ca^2+^ is approximately this value, ranging from 1.0 to 1.3 mm (45), although it can be substantially higher or lower depending on the local intra- or extracellular environment (46). Thus, Ca^2+^ binding to CD23 may play a critical role in a physiological setting. For example, the interactions between soluble trimeric CD23 and membrane IgE and between IgE-allergen complexes and membrane CD23 have been implicated in the regulation of IgE synthesis in B-cells (2, 9, 10); enhancement of the latter interaction by Ca^2+^ would also promote facilitated allergen presentation (11). Thus, the binding of Ca^2+^ brings an extra degree of modulation to CD23 function.

CD23 stands out among the Ig receptors as a member of a different superfamily, and although binding to human IgE does not require either carbohydrate or Ca^2+^, the latter does contribute to its activity, as we have seen, through binding at one of the CTLD's evolutionarily conserved sites. It is therefore particularly intriguing that, at least in human CD23, an amino acid side chain associated with binding a second Ca^2+^ ion in other CTLDs, Asp-258 (supplemental Fig. S8), has been conserved in CD23 and “co-opted” into making an additional interaction with IgE (Fig. 6*B*). In human CD23, the other auxiliary Ca^2+^ site residues have not been conserved, but in murine CD23 (and in several other species), the auxiliary site ligands *are* conserved, and binding of a second Ca^2+^ ion is predicted. It is unclear how this might affect IgE binding, but the effect of Ca^2+^ will certainly differ; indeed, the early report of a requirement for Ca^2+^ in the murine system is pertinent (16). Human CD23 differs from murine CD23 in other respects too, such as its interactions with CD21, which does not occur in the murine system (2). Many aspects of the role of CD23 in allergy may therefore be a relatively recent evolutionary development, but understanding its subtleties, such as the part played by Ca^2+^, may inform attempts to develop modulatory molecules for therapeutic purposes.

## Supplementary Material

Supplemental Data

## Footnotes

4 The abbreviations used are:

CTLD: C-type lectin-like domain
MBL: mannose-binding lectin
ITC: isothermal titration calorimetry
BisTris propane: 1,3-bis[tris(hydroxymethyl)methylamino]propane
PDB: Protein Data Bank.
