# Supplementary material for: Ca2+-dependent Structural Changes in the B-cell Receptor CD23 Increase Its Affinity for Human Immunoglobulin E
Source: J Biol Chem. 2013 Jun 17;288(30):21667–77. doi: 10.1074/jbc.M113.480657 (PMC3724626; doi:10.1074/jbc.M113.480657)
Supplement: Supplemental Data [file supp_288_30_21667__index.html]

Ca2+-dependent structural changes in the B-cell receptor CD23 increase its affinity for human Immunoglobulin E — Ca2+-dependent Structural Changes in the B-cell Receptor CD23 Increase Its Affinity for Human Immunoglobulin E — Ca2+-dependent Structural Changes in CD23 — Supplemental Data 

# Ca2+-dependent Structural Changes in the B-cell Receptor CD23 Increase Its Affinity for Human Immunoglobulin E

## 

**Files in this Data Supplement:**

- Supplementary Tables &#x26; Figures (.pdf, 6.3 MB) - Two tables and eight figures; descriptions for each are included in the file.
