# Supplementary material for: Ca2+-dependent Structural Changes in the B-cell Receptor CD23 Increase Its Affinity for Human Immunoglobulin E
Source: J Biol Chem. 2013 Jun 17;288(30):21667–77. doi: 10.1074/jbc.M113.480657 (PMC3724626; doi:10.1074/jbc.M113.480657)
Supplement: Supplemental Data [file supp_M113.480657_jbc.M113.480657-1.pdf]

SUPPLEMENTAL DATA

SUPPLEMENTAL FIGURE LEGENDS

Figure S1. Temperature dependence of IgE-Fc binding to derCD23 in the presence and absence of Ca^{2+}. (A) SPR binding curves for IgE-Fc binding to derCD23 in HBS and 4mM Ca^{2+} at 5°C (black), 15°C (red), 25°C (green) and 35°C (blue). (B) SPR binding curves for IgE-Fc binding to derCD23 in HBS and 10mM EDTA at 5°C (black), 15°C (red), 25°C (green) and 35°C (blue).

Figure S2. Conformation of Arg253 in the derCD23 structures. (A) Arg253 points away from the head domain in the Ca^{2+}-bound derCD23 structures (orange). (B) Arg253 projects into the cavity between loops 1 and 4 in conformation 2 of the Ca^{2+}-free derCD23 structures (cyan), with its guanidinium moiety forming a hydrogen bond with the main-chain carbonyl of Pro250.

Figure S3. Binding of Ca^{2+} to wild-type and mutant derCD23 measured by ITC. Binding isotherms of titrations of 20 mM $CaCl_2$ into 50 μM derCD23 at 25 °C together with a least-squares fit to the data, showing the heat absorbed per mole of Ca^{2+} *versus* the ratio of the total concentration of Ca^{2+} to the total concentration derCD23. (A) The wild-type derCD23 binding isotherm is shown. The wild-type, E249A and S252A fitted data are shown in black, red and green, respectively. (The results for mutants D258A and D270A are similar to that of E249A; data not shown for clarity.) (B) N269A mutant derCD23 binding isotherm and fitted data. (C) N269D mutant derCD23 binding isotherm and fitted data.

Figure S4. Superimposition of Ca^{2+}-bound and Ca^{2+}-free wild-type derCD23 structures and the four mutant structures. The colour coding for the various structures is indicated.

Figure S5. Ca^{2+}-binding site of the D258A mutant derCD23. Pro250 adopts the *trans* conformation, and the guanidinium group of Arg253 occupies the position of Ca^{2+} in the principal binding site.

Figure S6. Pre-incubation of derCD23 with cyclophilin A accelerates the observed rate constants for Ca^{2+} binding (red data points). The black data points are the results in the absence of Ca^{2+} re-shown for comparison. The green line shows the fit of the data in the presence of cyclophilin A (CycA) to a double exponential equation.

Figure S7. Superimposition of Ca^{2+}-bound and Ca^{2+}-free wild-type derCD23 structures, and the six Ca^{2+}-bound derCD23 structures in complex with Fcε3-4. The colour coding for the various structures is indicated. The uncomplexed derCD23 structures are as shown in Figure 2 (here lightly coloured), and superimposed are the six complexed derCD23 structures (in different shades of green).

Figure S8. Alignment of various CD23 and MBP sequences in the region containing the Ca^{2+} binding sites. The sequences have SwissProt IDs P06734, B8YM31, Q63097, P20693, F6Z682, E1BIQ4, P19999 and P08661 respectively, and are drawn with ESPript (1). Residues are numbered with reference to human CD23 and boxed according to an alignment produced with ClustalW2 (2); those strictly conserved have a red background and those well-conserved are indicated by red lettering. Secondary structural elements of human CD23 are indicated. Residues previously identified in rat MBP-A (3) as forming part of the principal Ca^{2+}-binding site (green diamond) or the auxiliary Ca^{2+}-binding site (orange star) are indicated.

Table S1. Primers for production of the derCD23 mutants.

DerCD23 mutation	Oligonucleotide sequences (5′→3′) Forward primer	Reverse primer
E249A	CAACTGGGCTCCAGGGGCGCCCACCAGCCGGAG	CTCCGGCTGGTGGGCGCCCCTGGAGCCGGAG
S252A	CCAGGGGAGCCCACCGCGCGGAGCCAGGGCGAG	CTCGCCCTGGCTCCGCGCGGTGGGCTCCCCTGG
D258A	CGGAGCCAGGGCGAGGCGTGCGTGATGATGCGG	CCGCATCATCACGCACGCCTCGCCCTGGCTCCG
N269A	GGCTCCGGTCGCTGGGCCGACGCCTTCTGCGAC	GTCGCAGAAGGCGTCGGCCCAGCGACCGGAGCC
N269D	GGCTCCGGTCGCTGGGACGACGCCTTCTGCGAC	GTCGCAGAAGGCGTCGTCCCAGCGACCGGAGCC
D270A	CTCCGGTCGCTGGAACGCGGCCTTCTGCGACCGTAAG	CTTACGGTCGCAGAAGGCCGCGTTCCAGCGACCGGAG

Table S2. Comparison of wild-type Ca^{2+}-bound and Ca^{2+}-free derCD23 structures.

	Ca^{2+}-A	Ca^{2+}-B	Ca^{2+}-C	Ca^{2+}-D	Apo-A	Apo-B	Apo-C	Apo-D
Ca^{2+}-A	-	0.49	0.84	0.83	0.81	0.93	1.17	1.20
Ca^{2+}-B		-	0.61	0.62	0.72	0.75	0.99	0.99
Ca^{2+}-C			-	0.49	0.67	0.56	0.87	0.89
Ca^{2+}-D				-	0.86	0.72	0.98	0.98
Apo-A					-	0.38	0.93	0.97
Apo-B						-	0.85	0.88
Apo-C							-	0.19
Apo-D								-

Wild-type Ca^{2+}-bound derCD23: Ca^{2+}-A, Ca^{2+}-B, Ca^{2+}-C and Ca^{2+}-D
Wild-type Ca^{2+}-free derCD23: Apo-A, Apo-B, Apo-C and Apo-D
Cα r.m.s. deviations (Å) are calculated over 120 Cα pairs.

Figure S1.

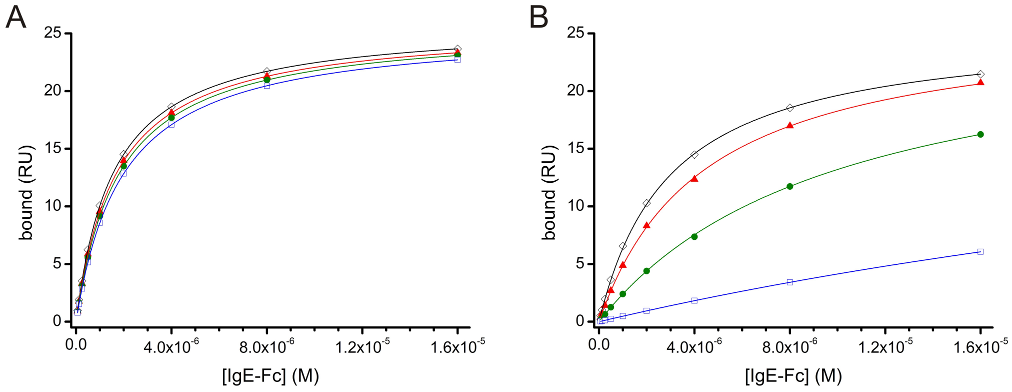

Figure S2.

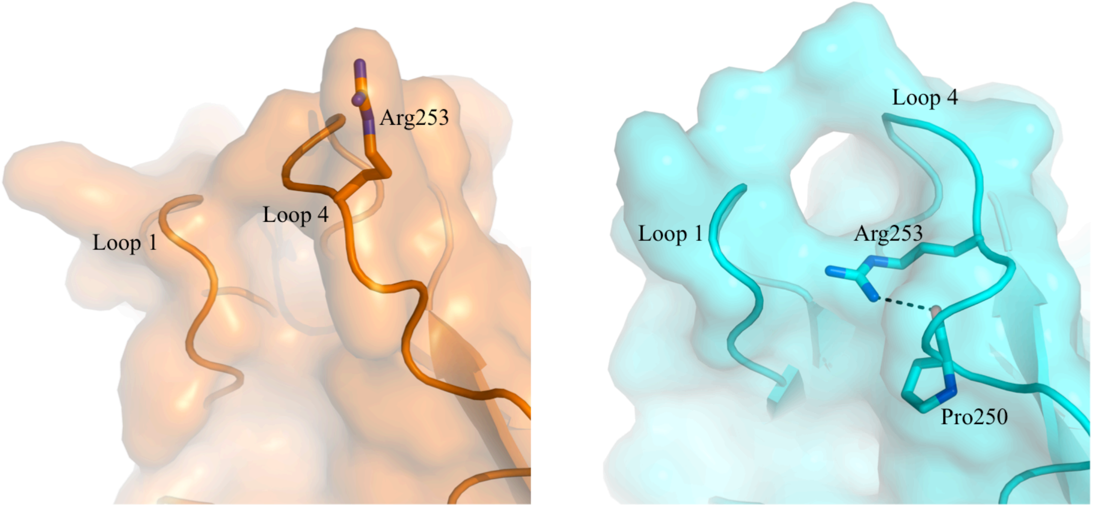

Figure S3.

(a)

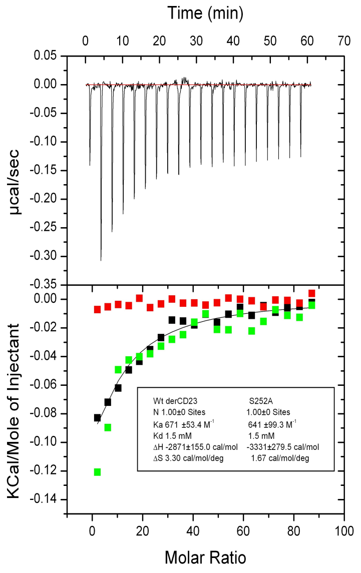

(b)

N269A

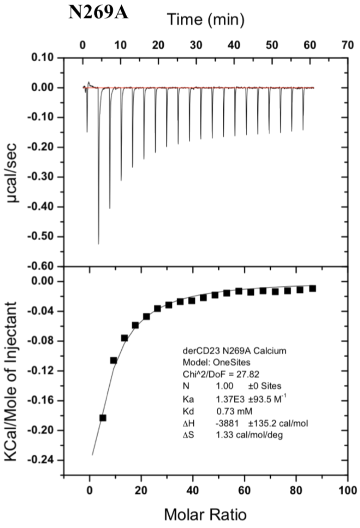

(c)

N269D

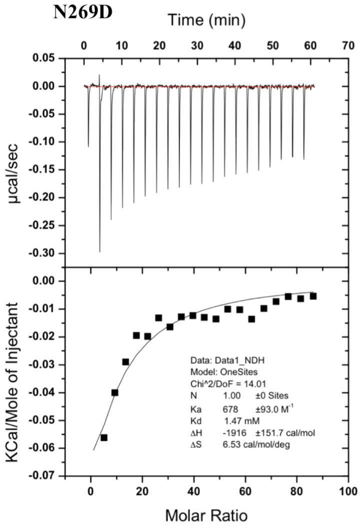

Figure S4.

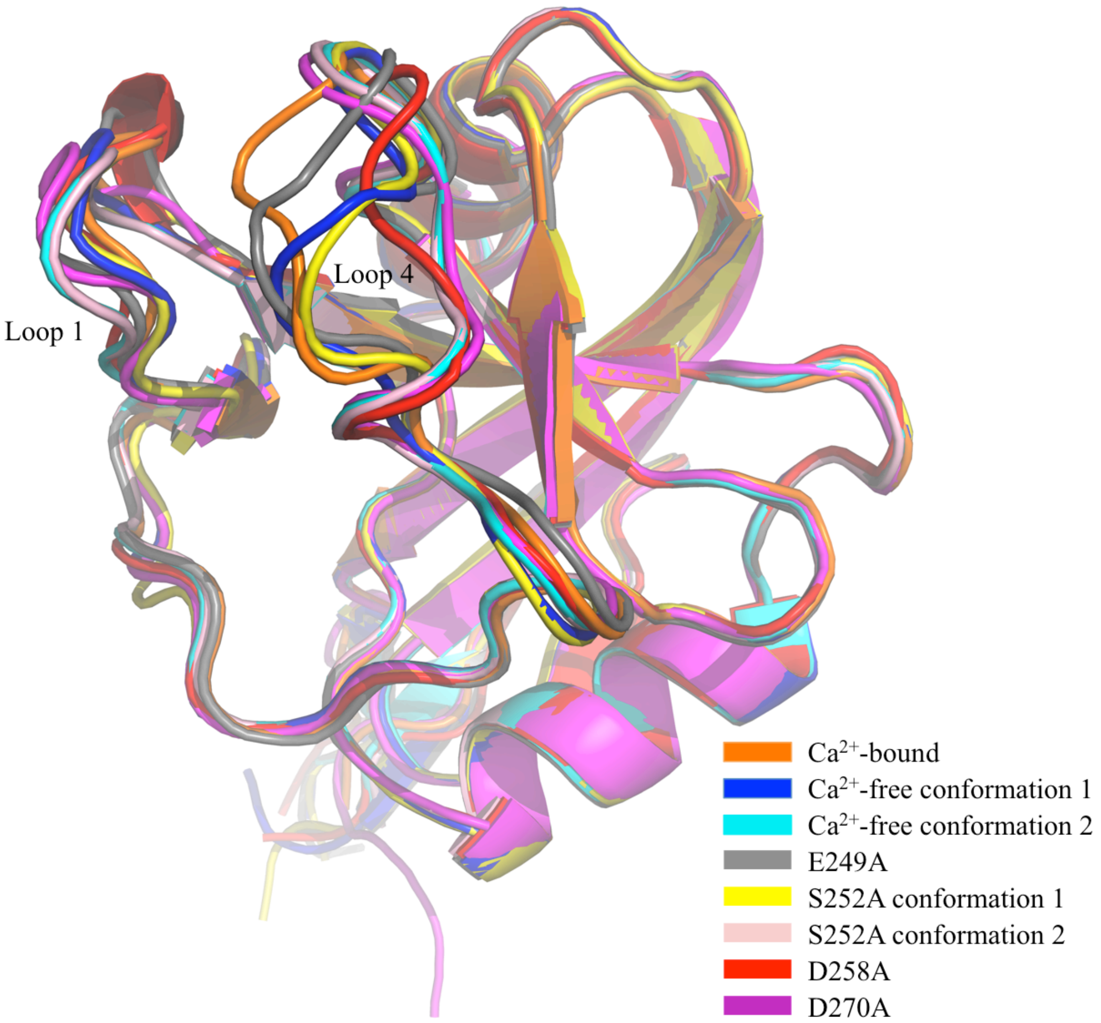

Loop 1
Loop 4
Ca2+-bound
Ca2+-free conformation 1
Ca2+-free conformation 2
E249A
S252A conformation 1
S252A conformation 2
D258A
D270A

Figure S5.

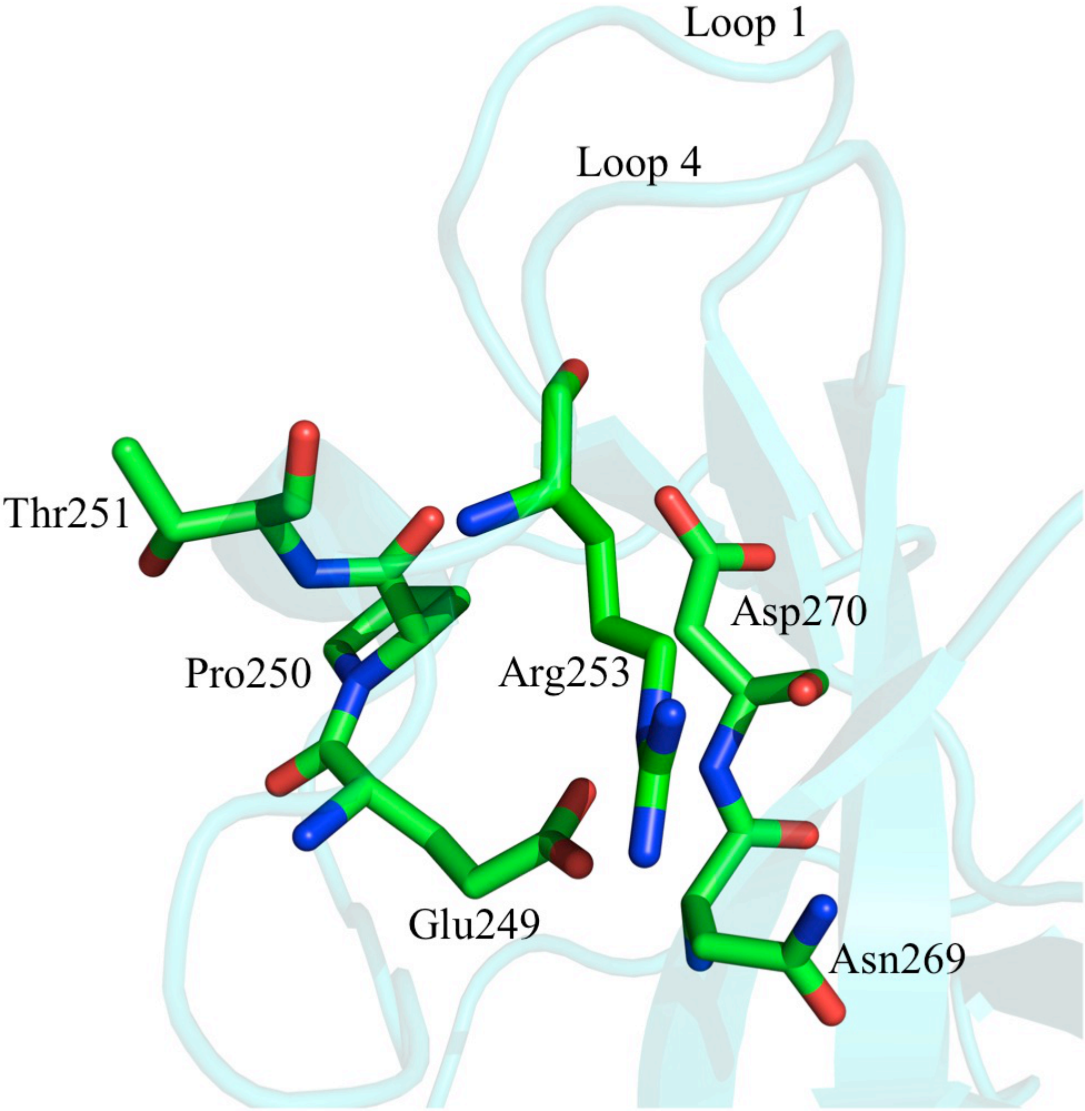

Figure S6.

Condition	k_{obs1} (s^{-1})	k_{obs2} (s^{-1})
-CycA	0.74	0.071
+CycA	0.92	0.104

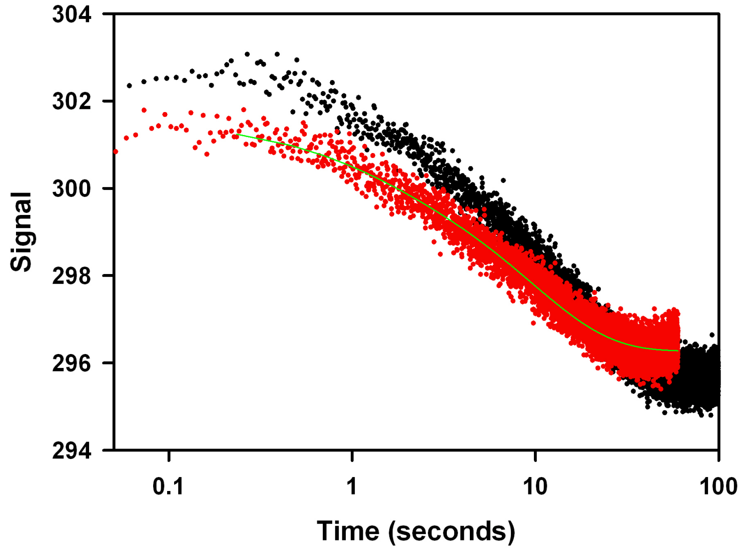

Figure S7.

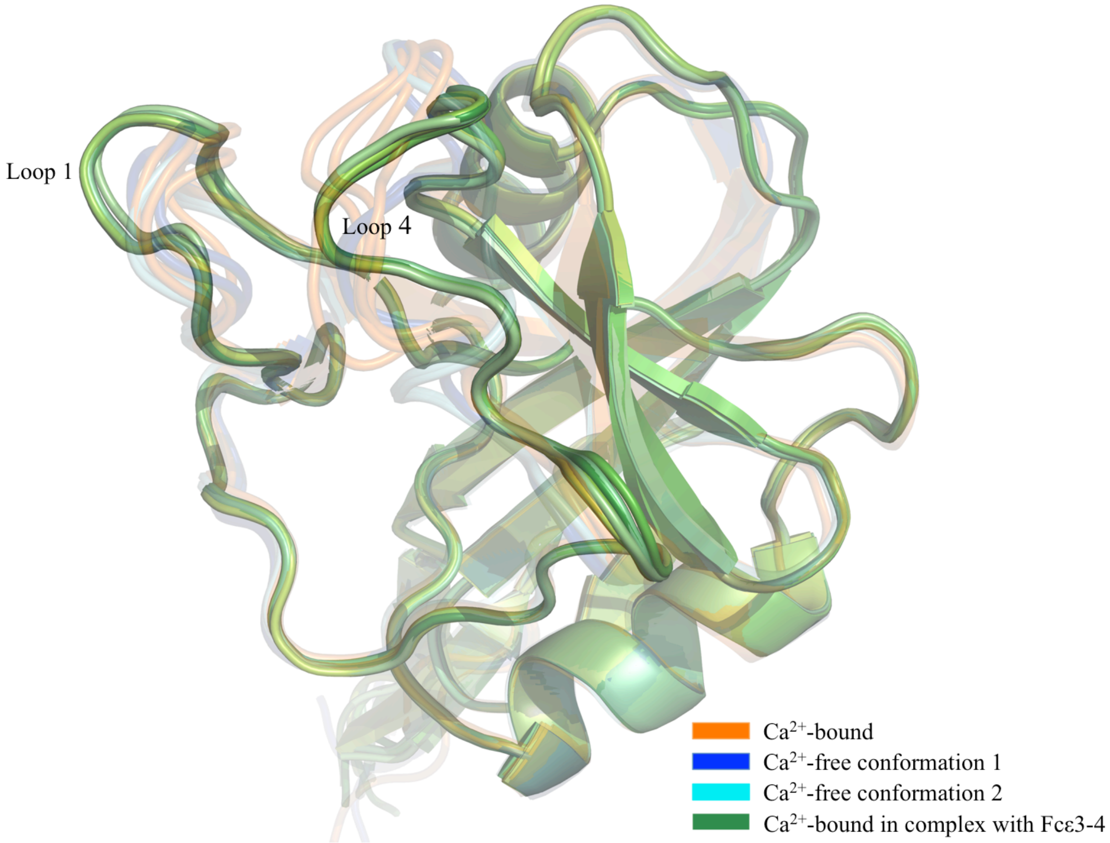

Loop 1
Loop 4
Ca2+-bound
Ca2+-free conformation 1
Ca2+-free conformation 2
Ca2+-bound in complex with Fcε3-4

Figure S8.

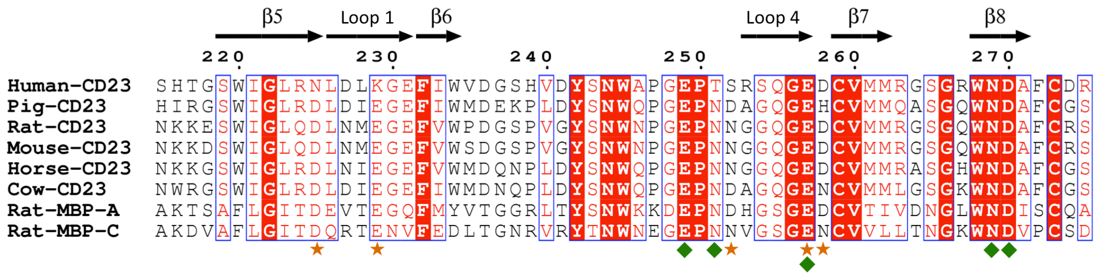

References

1. Gouet, P., Courcelle, E., Stuart, D. I., and Metoz, F. (1999) *Bioinformatics* **15**, 305-308
2. Thompson, J. D., Higgins, D. G., and Gibson, T. J. (1994) *Nucleic acids research* **22**, 4673-4680
3. Weis, W. I., Kahn, R., Fourme, R., Drickamer, K., and Hendrickson, W. A. (1991) *Science* **254**, 1608-1615
